# Research Progress on Phytochemicals from Mulberry with Neuroprotective Effects: A Review

**DOI:** 10.3390/ph18050695

**Published:** 2025-05-08

**Authors:** Junwei Chen, Zhonglang Gou, Yufei Huang, Qianhui Yu, An Na Kim, Wenchao Shi, You Zhou

**Affiliations:** 1 State Key Laboratory of Resource Insects, College of Sericulture, Textile and Biomass Sciences, Southwest University, Chongqing 400715, China; chenjunwei2567@outlook.com (J.C.); g18716650764@163.com (Z.G.); swcswu@swu.edu.cn (W.S.); 2 Westa College, Southwest University, Chongqing 400715, China; hwang_okbi@163.com (Y.H.); yqh837189@163.com (Q.Y.); 3 CDD Engine, Lino Lakes, MN 55014, USA; nnkimster@gmail.com

**Keywords:** mulberry, neurological disorders, phytochemicals, neuroprotection

## Abstract

With the intensification of the population aging worldwide, neurological disorders (NDs) are seriously threatening human society. Mulberry, a traditional economic crop, is a significant medicinal plant. Increasing evidence suggests that phytochemicals from mulberry play critical roles in the prevention and treatment of NDs. This paper reviews the recently reported phytochemicals from mulberry with neuroprotective effects and systematically summarizes neuroprotective mechanisms and their classifications. Based on their origins from different parts of mulberry, the extracts with neuroprotective effects are classified into mulberry fruit extract and mulberry leaf extract. According to the compound structures, the compounds are divided into flavonoids, Diels–Alder-type adducts (DAAs), benzofurans, quinones, stilbenes, and alkaloids. This aims to provide a future reference for their pharmaceutical development and utilization.

## 1. Introduction

**Figure 1:**
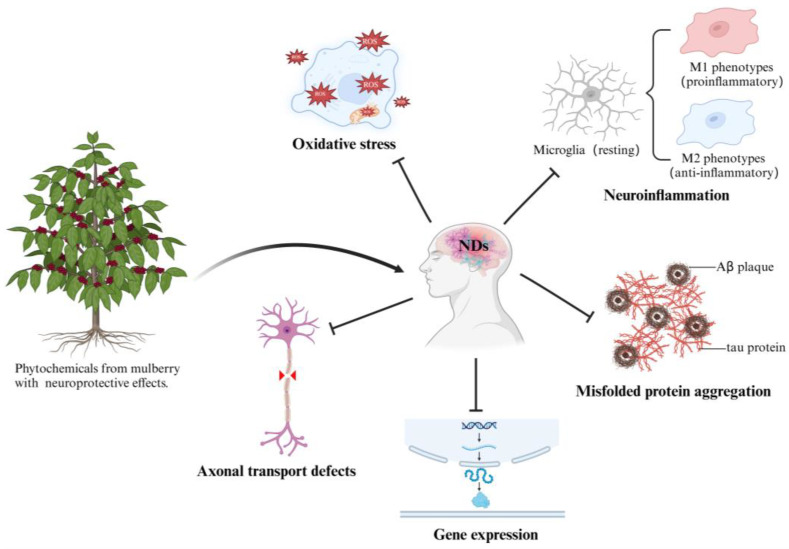
The five major pathological mechanisms of NDs and the multi-target neuroprotective effects of mulberry phytochemicals. Created in Biorender. Junwei Chen (2025) https://BioRender.com.

Neurological disorders (NDs) are diseases associated with the central and peripheral nervous system [1]. NDs can be categorized into three main types: (a) non-communicable diseases, (b) communicable and other diseases, and (c) injuries. Non-communicable diseases include neurodegenerative diseases (NDDs), strokes, idiopathic epilepsy, migraines, brain cancers, motor neuron diseases, and down syndrome; communicable and other diseases include neonatal encephalopathy caused by birth asphyxia and trauma, meningitis, encephalitis, tetanus; injuries involve head injuries and spinal injuries [2]. Notably, with the growth of the global population and the intensification of the aging trend, approximately 3.4 billion people worldwide suffer from NDs, accounting for 43% of the global population [3]. This represents a significant burden on global health systems. Despite significant advancements in various surgical, pharmacological, and interventional treatments in recent years, most patients with NDs are only clinically diagnosed when the nerve damage becomes severe, leading to the loss of appropriate timing for treatment, and the prognosis of patients remains unfavorable overall [4]. The pathological progression of NDs involves multiple mechanisms, including but not limited to oxidative stress, neuroinflammation, misfolded protein aggregation, gene expression, and axonal transport defects (Figure 1). Among many mechanisms, brain damage caused by oxidative stress is one of the main pathogenic factors of such diseases leading to the development process of NDs [5,6]. The generation and clearance of reactive oxygen species (ROS) in the body are dynamically regulated under normal physiological conditions. However, under pathological conditions, such as NDDs or acute brain injury, abnormalities in the electron transport chain or excessive inflammatory responses are triggered. The ROS production rate significantly exceeds the scavenging capacity of the antioxidant system, leading to an imbalance in redox homeostasis. Excess ROS can damage the structure and function of intracellular biomacromolecules, such as injuring cell membranes, denaturing proteins, and disrupting nucleic acid structures, thereby affecting the normal physiological functions of nerve cells and causing oxidative damage to the central nervous system (CNS). Furthermore, ROS can also directly bind to the mitochondrial membrane and alter its structure and function. Continuous mitochondrial damage will further induce chronic inflammation, aggravating and even leading to the death of neuronal cells [7,8]. Neuroinflammation is also an important segment in the pathological progression of NDs. Under such pathological conditions the inherent neuroimmune cells in the CNS-microglia are activated and then differentiated into two types, the classic proinflammatory M1 and the anti-inflammatory M2 phenotypes. Although the inflammatory response is essential for the body to resist external invasion to a certain extent, a continuous neuroinflammatory response may cause damage to nerve cells, affect the signal transmission between nerve cells and result in the loss of neurons in the CNS [9]. The aggregation of misfolded proteins within nerve cells is another feature of NDs, such as β-amyloid protein (Aβ), phosphorylated tau protein (p-tau), α-synuclein protein (α-Syn), and huntingtin protein, as common inducing factors and markers of NDDs [10]. Aβ is a key factor in the pathology of Alzheimer’s diseases (AD). It is a peptide produced upon the cleavage of the large type I transmembrane protein amyloid precursor protein (APP) in neurons. In AD patients, the 42-residue variant of the Aβ peptide (Aβ_42_) can undergo progressive aggregation during amyloidosis to form dimers, oligomers, and protofibrils. These aggregations are cytotoxic and can affect the normal metabolism and physiological functions of nerve cells, triggering the apoptosis of nerve cells [11,12]. Axons are an important component of nerve cells, responsible for transporting substances synthesized in the cells to the axon terminals, and simultaneously transporting substances taken up at the axon terminals back to the cells. Many NDs share a common feature: the disruption of axonal transport leading to the obstruction of the nutrient supply to nerve cells, abnormal synthesis and release of neurotransmitters, and the degeneration and death of nerve cells [13]. Moreover, recent scientific research has revealed the crucial role of gene mutations and expressions in the pathogenesis of NDs [14]. Mutations in specific genes can result in abnormal structures or functions of the encoded proteins, thereby directly or indirectly compromising the normal development, survival, and functional maintenance of nerve cells [15]. Studies have demonstrated that individuals carrying specific genes exhibit significantly elevated risks of developing NDDs: for instance, the APP gene carried by patients with AD [16], and the α-synuclein (SNCA) gene carried by patients with Parkinson’s diseases (PD) [17]. It is, therefore, important to avoid irreversible CNS damage caused by oxidative stress, neuroinflammation, and misfolded proteins aggregation for preventing the occurrence of NDs and slowing down the progression of the patient’s symptoms.

**Figure 2:**
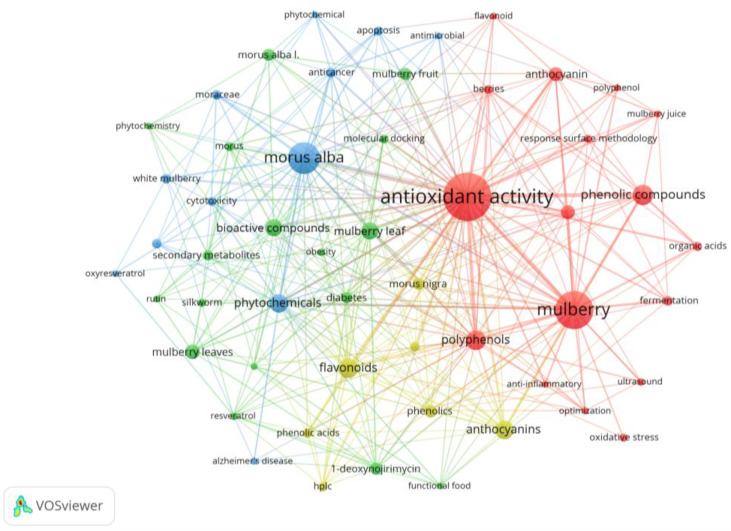
Keyword co-occurrence network for the research on phytochemicals from mulberry based on VOSviewer (2010–2024). Data Source: Web of Science Core Collection (Search Formula: TS = (“Morus alba” OR “Mulberry” OR “mulberry tree”) AND TS = (phytochemical* OR phytoactive* or secondary metabolite* OR bioactive compound*), with 747 studies included. Identify a total of 53 different keywords with seven or more occurrences.

In view of the multifactorial pathogenesis of NDs, the development of multi-target, synergistically modulated therapeutic approaches has increasingly become an important trend in the field [18]. Natural phytochemicals have attracted much attention due to their structural diversity and promising bioactivities [19,20], with their neuroprotective effects validated across multiple models. For example, the ethanol extract of *Scoparia dulcis* significantly improves memory impairment in zebrafish models by reversing acrylamide-induced neuronal degeneration and behavioral dysfunction [21]. Berberine, a natural alkaloid primarily isolated from *Coptis chinensis* and *Berberis vulgaris*, has demonstrated multiple protective effects in ischemic stroke models, including the promotion of thrombolysis and the inhibition of oxidative stress and inflammatory responses, resulting in reducing brain infarct damage [22]. However, some phytochemicals still encounter obstacles in practical applications, including high toxicity and single mechanism of action. For instance, celastrol, a pentacyclic triterpenoid isolated from *Tripterygium wilfordii*, demonstrates therapeutic efficacy in NDDs via nuclear factor-kappa B (NF-κB) and heat shock proteins (HSPs) inhibition, but its narrow therapeutic window together with the occurrence of adverse effects have hindered further research [23,24]. The latest reported phytochemicals with neuroprotective effects exhibit a principal biological activity: quercetin as an antioxidant, cannabidiol as an anti-inflammatory, and N, N-dimethyltryptamine as a promoter of neuroplasticity [25]. They are unable to effectively intervene in the disease’s progression. These limitations emphasize the need to develop more phytochemicals that combine multiple mechanisms of action with a favorable safety profile. Mulberry of the genus *Morus* of the family Moraceae is not only a traditional economic crop but also an important medicinal plant. Traditional Chinese medical classics, such as “*Compendium of Materia Medica*” and “*Shennong’s Classic of Materia Medica*”, have numerous records regarding the medicinal activity of mulberry leaf and often associate mulberry leaf with longevity. Modern studies have indicated that the mulberry branch, leaf, fruit, and root bark are rich in alkaloids, flavonoids, polysaccharides, and other chemical components, and exhibit anti-tumor, anti-microbial, immunoregulatory, and other pharmacological activities [26,27]. By analyzing the bibliometric of the last 15 years within the field of mulberry phytochemicals using the VOSviewer, the result (Figure 2) reconfirmed that the phytochemicals have a wide range of bioactivities including anti-cancer, anti-inflammatory, and antimicrobial. In Figure 2, color represents cluster, node size refers to frequency of appearance, and link thickness represents co-occurrence intensity. Obviously, among the bioactivities possessed by phytochemicals from mulberry, “antioxidant activity” occupies the most important place. This implies a strong relationship between mulberry phytochemicals and oxidative stress, the core pathological mechanism of NDs. Increasing evidence highlights phytochemicals from mulberry exert neuroprotective effects through multiple mechanisms of action such as anti-oxidation, anti-neuroinflammation, and the regulation of gene expression (Figure 1). In this review, we systematically summarize recent advances in pharmaceutical research and therapeutic applications of phytochemicals from mulberry for updated knowledge about its neuroprotective effects.

## 2. Mulberry Extracts with Neuroprotective Effects

### 2.1. Differences Between Mulberry Leaf and Fruit Extracts

As different medicinal parts of the mulberry, the leaf and fruit have similar bioactivities but with different focuses (Table 1). Studies have shown that mulberry leaf extract shows unique advantages in metabolic regulation, with its anti-diabetic and hypolipidemic effects being particularly prominent [28,29]. In contrast, mulberry fruit extract exhibits predominant antioxidant and anti-inflammatory activities [26]. This discrepancy is attributed to their characteristic phytochemicals. The key bioactive constituents in mulberry leaf consist of flavonoids such as quercetin and rutin, along with alkaloids represented by 1-deoxynojirimycin (DNJ, an α-glucosidase inhibitor) [30]. The efficacy of mulberry fruit mainly relies on anthocyanins dominated by cyanidin-3-glucoside (C3G) and phenolic acids represented by hydroxycinnamic acid derivatives [26]. In the extraction process, due to the dense fibrous tissue of mulberry leaf, cellulase pretreatment or ultrasound-assisted cell wall disruption is often required to improve extraction efficiency [31,32]. Although the cell wall of fruit is relatively thin (water content > 80%) and can be broken by conventional homogenization, the inherent instability of anthocyanins in fruit requires strict light avoidance environments and temperature-controlled conditions throughout extraction [33]. Notably, both cultivar selection and harvest timing critically influence target component concentrations. For instance, the content of DNJ in the leaf of *Morus australis* (1.01 mg/g) is 40% higher than that in *Morus alba* [34], while the total anthocyanins content in the fruit of *Morus nigra* (185 mg/100 g) can reach 6.6 times that of *Morus alba* [35]. In terms of seasonal changes in content, DNJ level usually starts to increase in April, peaks in June or July, then gradually decreases, and remains at a relatively low level from September to October [36]. The content of anthocyanins in fruit gradually increases during the ripening process, being almost undetectable in unripe fruit and reaching a peak at the fully mature stage [37]. These differences suggest that researchers should select appropriate cultivars, optimal harvest period, and tailored extraction protocols based on target bioactive ingredients. It is noteworthy that despite these differences, both mulberry leaf and fruit extracts can achieve neuroprotection through mechanisms including alleviating oxidative stress and inhibiting toxic protein aggregation, and their neuroprotective effects will be described in detail below.

### 2.2. Extract of Mulberry Fruit

The extract of mulberry fruit is abundant in anthocyanins, phenolic acids, polyphenols, and many other chemical components. It exhibits multiple beneficial effects including antioxidant, immunomodulatory, antitumor, hypolipidemic activities, and so on [26]. For instance, the combined treatment of mulberry fruit water extract and paclitaxel significantly inhibits the growth of bladder cancer tumors, and the inhibitory effect of this drug combination on cancer is greater than either drug alone [38]. Recent studies show the extract of mulberry fruit is also a natural cognitive enhancer and neuroprotectant against NDs.

The mulberry fruit extract manifested a protective effect on brain injury and memory impairment in rats. Kaewkaen and colleagues [39] dried mulberry fruit and ground them to powder, then extracted the powder with ethanol. The neuroprotective effects of mulberry extract were verified using a middle cerebral artery occlusion (MCAO) rat model. Its mechanisms of action involve reducing oxidative stress and inhibiting apoptosis [39]. The MCAO mice treated with mulberry fruit extract showed significant changes in oxidative stress markers, including reduced level of malondialdehyde (MDA) and increased levels of superoxide dismutase (SOD) and glutathione peroxidase (GSH-Px). Meanwhile, Bcl-2-immunopositive neurons density was found to be increased, contributing to the inhibition of neuronal apoptosis in rat hippocampus. These changes resulted in an increase in the density of neurons in the hippocampus, especially in the CA3 region. This enhances the encoding and retrieval efficiency during the learning and memory processes, consequently ameliorating memory impairment. Liu and colleagues [40] obtained ethanol extract of mulberry fruit by using a method similar to that of Kaewkaen [39]. After treatment with mulberry fruit extract (100 milligram per kilogram body weight, mg·kg^−1^·b.w.), the level of Aβ plaques in the brain tissue of APP/PS1 double transgenic mice was significantly decreased, showing a positive impact on preventing the early onset of AD [40]. Meanwhile, treatment with mulberry fruit extract in APP/PS1 mice significantly increased NeuN^+^ cell density (indicating viable neurons, including cerebral pyramidal neurons, granular neurons, and cerebellar granule cells) while reducing TUNEL^+^ cell counts (detecting DNA fragmentation in apoptotic cells). This result demonstrated effective suppression of cortical and hippocampal neuronal apoptosis. In addition, the mulberry fruit extract significantly reduced the levels of inflammatory factors such as IL-1β, IL-6, and TNF-α measured by the enzyme-linked immunosorbent assay (ELISA) method, thereby effectively alleviating the stress response of neuroinflammation. Notably, the therapeutic effects of the above studies were all superior to those of the mice subjected to donepezil (a standard drug for the dementia treatment and used as a positive control) [40]. The brain dysfunctions of APP/PS1 mice, including spatial learning and memory abilities, were significantly improved.

Mulberry fruit extract exhibits neuroprotective effects in the AD model by inhibiting the cytotoxicity caused by oxidative stress and Aβ aggregation. Yang and colleagues [41] established an AD model by treating rat adrenal pheochromocytoma cells (PC12) with Aβ (20 μmol·L^−1^). Phenolic extract was obtained from mature mulberry fruit through a complex extraction process. The results showed that, compared with negative control (treated only with Aβ), the viability of PC12 cells treated with phenolic extract (200 μg·mL^−1^) increased by 49%. Further research revealed that the extract could maintain the activity of SOD and neutralize ROS, consequently alleviating oxidative stress. In the same AD model, it was also found that the mulberry fruit extract without amino acids and vitamins could still significantly restrain the death of PC12 cells [42]. These results indicate that the phenolic active components in mulberry fruit may mainly exert neuroprotective effects. Ochiishi and colleagues [43] reported that mulberry fruit ethanol extract (250 μg·mL^−1^) significantly increased the fluorescence level of human embryonic kidney cells (HEK293T) samples in Aβ aggregation assay using the green fluorescent protein (GFP) fluorescent labeling method. This suggested that Aβ aggregate was dissociated to a certain extent after treatment with mulberry fruit extract, and the microenvironment around the GFP chromophore was restored to a state favorable for fluorescence emission, exhibiting promising neuroprotective effects.

It was also reported that mulberry fruit extract could exert a beneficial effect on PD by lowering the ROS level in nerve cells. A PD model was established by exposing human neuroblastoma cells (SH-SY5Y) to 6-hydroxydopamine (6-OHDA). It was found that mulberry fruit ethanol extract (0.1–100 μg·mL^−1^) could increase the viability of SH-SY5Y cells and significantly inhibit the generation of ROS and NO at doses of 10 and 100 μg·mL^−1^, consequently reducing the damage caused by oxidative stress [44].

Taken together, the mulberry fruit extracts exert neuroprotective effects through multiple pathways and levels, mainly by reducing the level of ROS in nerve cells, inhibiting the aggregation of Aβ peptides, suppressing the occurrence of neuroinflammation and apoptosis, which are all dependent on the abundance of natural bioactive compounds in mulberry fruit extract.

### 2.3. Extract of Mulberry Leaf

Mulberry leaf exhibits versatile applications and holds an important position in silk manufacturing, silkworm rearing, and the food industry. Mulberry leaf extract is rich in phenols, alkaloids, flavonoids, etc., and exhibits diverse biological activities. As a traditional Chinese herbal medicine, mulberry leaf is commonly utilized in the treatment of neurological disorders such as cerebral hemorrhage and ischemia [45]. The neuroprotective mechanisms of mulberry leaf extract have been studied and elucidated to some extent.

Mulberry leaf extract exerts neuroprotective effects in different experimental models by alleviating the damage caused by oxidative stress. Hamdan and colleagues [46] extracted mulberry leaf with 80% ethanol and took the ethyl acetate fraction of leaf (ET-L). It was found that ET-L (200 mg·kg^−1^·b.w.) could significantly increase the levels of various amino acids (glutamic acid, aspartic acid, serine, and histidine, etc.) in mice brains treated with cisplatin (10 mg·kg^−1^·b.w.). It also regulated the levels of oxidative stress markers, including reducing the levels of MDA and 8-hydroxy-2′-deoxyguanosine (8OHdG) and increasing the level of reduced glutathione (GSH). In another model where glyphosate was used to induce neurotoxicity in mice, it was found that mulberry leaf extract could significantly reduce the level of lactate dehydrogenase (LDH) and increase the activity of SOD in the mice brains. This indicates that mulberry leaf extract can alleviate the damage caused by oxidative stress, reduce the level of ROS in the brain, and exert neuroprotective effects [47]. Kang and colleagues [48] established experimental models of oxidative damage and cerebral ischemic cytotoxicity by exposing PC12 cells to H_2_O_2_ and oxygen–glucose deprivation (OGD), respectively. It was found that the mulberry leaf extract could reduce the mortality of PC12 cells. They suggested that the strength of the neuroprotective activity might be correlated with the content of γ-aminobutyric acid (GABA) in the mulberry leaf extract.

Similar to the neuroprotective mechanisms of action of mulberry fruit extract, the mulberry leaf extract can also inhibit Aβ self-aggregation. Niidome and colleagues [49] obtained the mulberry leaf methanol extract by treating mulberry leaf with methanol at 55 °C for 5 h followed by centrifugation. Using the fluorescent dye thioflavin T (ThT) to label Aβ fibrils, it was found that the methanol extract induced a dose-dependent reduction in ThT fluorescence intensity and significantly shortened Aβ fibril length observed under electron microscope. These findings suggest that the methanol extract would effectively restrain the Aβ_42_ formation. Further research found that mulberry leaf extract also dose-dependently reduced cytotoxicity and effectively maintained the physiological morphology of mouse hippocampal neurons in a Aβ_42_-induced toxicity test.

It has been reported that mulberry leaf extract also has an antidepressant-like effect. The concentration of glutamate in the brains of patients with depression is relatively high. Glutamate, a classical excitatory neurotransmitter in the CNS, can induce neuronal cell death through excitotoxicity and oxidative stress when its concentration is excessive. According to existing reports, the oxidative stress mediated by glutamate is mainly attributed to the inhibition of cysteine uptake, which leads to a significant decrease in intracellular glutathione content and accumulation of ROS [50,51]. Dalmagro and colleagues [52] reported that oral administration of the black mulberry leaf aqueous extract could dose-dependently inhibit the cell death induced by glutamate in the hippocampal and cortical slices of mice. This effect might be associated with the syringic acid, the most abundant compound in the aqueous extract (accounting for 80%), but the specific mechanism remains to be further elucidated.

Compared with mulberry fruit and mulberry leaf extracts, the neuroprotective effects and mechanisms of extracts from other parts of mulberry have not yet been reported. For the reported active extracts, it is particularly crucial in exploring further the compounds with neuroprotective effects. Identifying these compounds is helpful for not only understanding in depth the intrinsic mechanism of the neuroprotective effects exerted by mulberry but also providing medicinal chemists with insights for developing novel neuroprotective agents, promoting the practical application of mulberry in the field of neuroprotection.

## 3. Chemical Components of Mulberry with Neuroprotective Effects

The chemical components with neuroprotective effects in mulberry are mainly classified as flavonoids, Diels–Alder-type adducts, benzofurans, quinones, stilbenes, and alkaloids.

### 3.1. Flavonoids

Flavonoids mainly exist in natural plants and have a wide range of biological activities. Based on the existing reports, flavonoids with neuroprotective effects isolated from mulberry can be classified into flavonols, dihydroflavones, anthocyanidins, and chalcones.

#### 3.1.1. Flavonols

**Figure 3:**
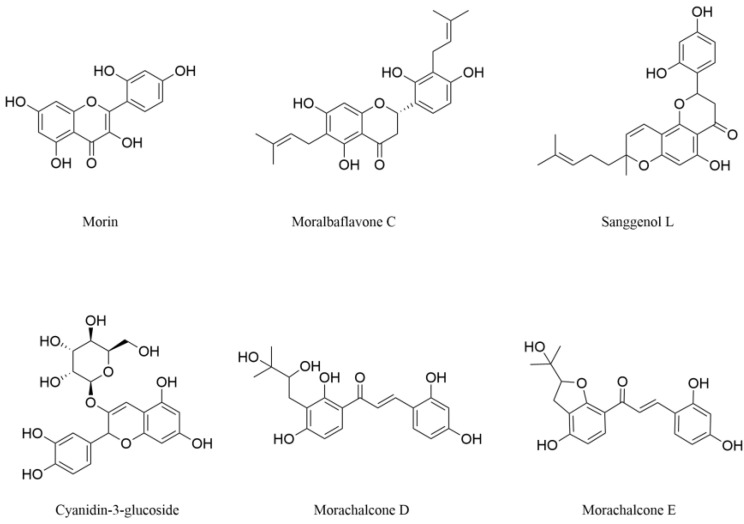
The structures of flavonoids from mulberry with neuroprotective effects.

Flavonols, structurally defined as 3-hydroxyflavones, constitute a principal subclass of flavonoids ubiquitously distributed in plants. They exhibit various biological activities, including antioxidant, antibacterial, hepatoprotective, and anti-inflammatory activity [53]. Morin (Figure 3), a typical flavonol compound, exerts beneficial effects by reducing oxidative stress in mice with nerve damage [54]. Mice subjected to sleep deprivation showed a decrease in GSH concentration, and a significant increase in MDA (an index of lipid peroxidation) and nitrite concentration (an indicator of nitric oxide production in the brain), indicating the occurrence of oxidative stress in the mice and resulting in hippocampal damage. It was reported that within the tested dose range (5–20 mg·kg^−1^·b.w.), morin hydrate could reverse these changes, significantly improve the cognitive ability of mice, and reduce anxiety-like behaviors [54]. In addition, morin also shows potential in the treatment of PD. Early studies have confirmed that morin can exert neuroprotective effects by inhibiting the activation of astrocytes and the nuclear translocation of NF-κB induced by 1-methyl-4-phenyl-1,2,3,6-tetrahydropyridine (MPTP, a pro-neurotoxin) [55]. However, the precise molecular mechanisms underlying these neuroprotective effects remained elusive until 2023, when Wang and colleagues [56] first elucidated that morin improved PD pathology through a dual-action mechanism: it activated the AMPK-ULK1 signaling pathway and enhanced nuclear translocation of transcription factor EB (TFEB). These coordinated actions induced mitophagy, amplified autophagy flux, and mitigated inflammation-mediated neuronal apoptosis. Unlike conventional mitophagy inducers that disrupt mitochondrial membrane potential or inhibit respiratory chain activity [57], morin can maintain the low mitochondrial membrane potential (a necessary condition for mitophagy) while preserving basal mitochondrial homeostasis. This unique property significantly reduces potential side effects. Furthermore, in MPTP-induced PD mouse models, 14-day oral administration of morin (25 mg/kg) significantly ameliorated motor ability deficits (evidenced by prolonged latency in rotarod tests) and exploratory behavior impairments (reflected by increased locomotion distance in open-field assays). Notably, the mouse effective dose corresponds to a human equivalent dose of 2 mg/kg—a clinically achievable threshold [56]. These findings not only clarify the unique mechanism of action of morin but also provide crucial evidence for the translation of morin from laboratory research to clinical application.

#### 3.1.2. Dihydroflavones

Dihydroflavones are isolated from the root bark of mulberry. They mainly exert neuroprotective effects by alleviating oxidative stress. Moralbaflavone C (Figure 3) is a prenylated flavonoid isolated by Zhu and colleagues [58]. They found that moralbaflavone C (10 μmol·L^−1^) could significantly alleviate the oxidative damage of PC12 cells induced by sodium nitroprusside (SNP). It increased cell viability more remarkably than the positive control edaravone at the same concentration, exhibiting a promising protective effect.

Sanggenol L (SL, Figure 3) has been reported to possess many biological activities including anti-cancer, anti-inflammatory, and neuroprotective [59,60,61]. Zhao and colleagues [62] used rotenone-stimulated human neuroblastoma cells SK-N-SH to establish an in vitro model of PD. It was found that SL could alleviate mitochondrial dysfunction and ROS production by regulating apoptotic proteins, promoting the proliferation of SK-N-SH cells treated with rotenone. Furthermore, SL also dose-dependently reversed the down-regulation of phosphorylation levels of PI3K, Akt, and mTOR in rotenone-induced SK-N-SH cells. Reactivating this signaling pathway can inhibit cell apoptosis and promote the stimulation of autophagy, which clears damaged mitochondria and protein aggregates, thus protecting cells from oxidative stress damage [62].

#### 3.1.3. Anthocyanins

C3G (Figure 3) is a precursor for anthocyanin synthesis. It possesses free radical scavenging and inflammation suppressing activities, and provides protection to endothelial dysfunction [63,64]. C3G achieves neuroprotective activity by maintaining the normal function of mitochondria, and its mechanisms of action have been gradually revealed in the following studies. As early as 2006, Kang and colleagues [65] used the mice model of MCAO and confirmed that 1% HCl-methanol mulberry fruit extract had protective and improvement effects on brain obstruction and brain ischemia-induced damages, and found that C3G was the dominant component with neuroprotective effects in the extract. From the assessment of the neuroprotective activity of C3G, it was found that C3G (10–30 μg·mL^−1^) dose-dependently increased the viability of PC12 cells exposed to H_2_O_2_ [65]. In 2011, the study by Bhuiyan and colleagues [66] reported that by inducing rat cortical neuron cells with glutamate to simulate neurotoxicity, there was no significant disparity in the cell mortality rate between the cells treated with C3G and the group treated only with glutamate. However, in cerebral ischemia induced by OGD, C3G (1–10 μg·mL^−1^) significantly increased the survival rate of rat cortical neurons in a dose-dependent manner and reduced the release of LDH. Furthermore, the intensity of neuronal mitochondrial membrane potential (MMP) was used as a measurement index to reflect whether mitochondrial function was normal. It was found that OGD injury induced a severe mitochondrial depolarization in rat cortical neurons, as quantified by the fluorescent dye rhodamine-123. The MMP was assessed by calculating the fluorescence intensity ratios of treated neurons to untreated controls. OGD insult reduced the MMP signal intensity to 26%, indicating profound mitochondrial dysfunction. Treatment with C3G at doses of 5 and 10 μg·mL^−1^ restored MMP signal intensity to 59% [66]. Thus, it can be concluded that C3G in mulberry exerts neuroprotective activity by maintaining the normal function of mitochondria in rat cortical neurons, rather than by reducing glutamate-induced neurotoxicity.

#### 3.1.4. Chalcones

Chalcones are a class of open-chain flavonoids characterized by two aromatic rings linked through an α, β-unsaturated ketone system [67]. Chalcones can scavenge ROS through redox active phenolic hydroxyl groups and inhibit the neuroinflammatory response mediated by NF-κB [68,69]. These properties endow them with neuroprotective potential. Two novel chalcones, morachalcone D and morachalcone E, isolated from mulberry leaf exhibit significant neuroprotective effects through dual mechanisms of anti-oxidative stress and anti-ferroptosis. Their structures are shown in Figure 3. Wen and colleagues [70] used the endogenous oxidative damage in mouse hippocampus-derived HT22 cells induced by glutamate and ferroptosis inducer erastin as models to explore the neuroprotective effects of the two compounds. Ferroptosis is a novel form of cell death that is initiated by intracellular iron (Fe^2+^) overload and excessive accumulation of lipid peroxides, causing damage to the cell membrane and organelle membranes, thereby leading to cell death [71]. It was found that two compounds dose-dependently increased the viability of HT22 cells in both models, but the neuroprotective effects of morachalcone D were superior to morachalcone E. To delineate the molecular basis of morachalcone D-mediated neuroprotection, they analyzed oxidative stress markers and associated regulatory networks in HT22 cells. The results showed that morachalcone D (20–40 μmol·L^−1^) significantly and dose-dependently reduced ROS content, increased GSH content, and decreased the accumulation of Fe^2+^. Mechanistically, this compound activated a multi-layered antioxidant defense system through the upregulation of the nuclear factor erythroid 2-related factor 2 (Nrf2) signaling pathway. It enhanced the expression of core redox enzymes including glutathione peroxidase 4 (GPx4), catalase (CAT), SOD, and Nrf2-regulated auxiliary components including heme oxygenase-1 (HMOX1) and solute carrier family 7 member 11 (SLC7A11) [70]. The results suggest that morachalcone D may be an effective neuroprotective agent.

### 3.2. Diels-Alder-Type Adducts

**Figure 4:**
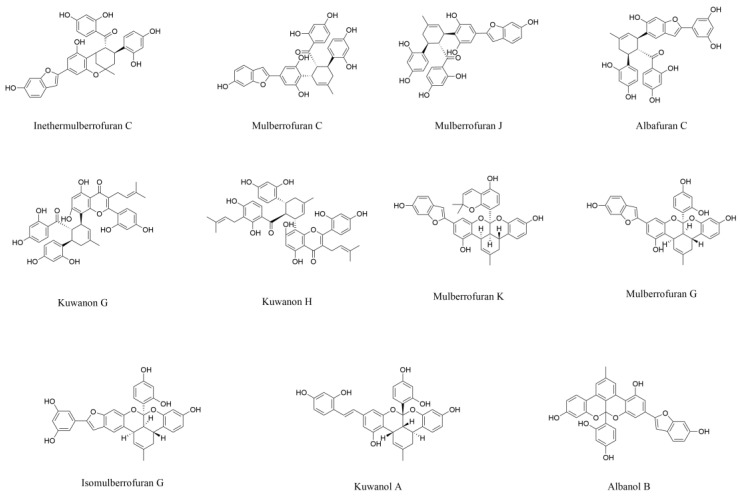
The structures of Diels–Alder-type adducts from mulberry with neuroprotective effects.

Diels–Alder-type adducts (DAAs), a class of structurally distinct phenolic compounds characteristic of *Morus* plants, are biosynthesized through an intermolecular [4+2] cycloaddition of chalcones and dehydroprenylphenols [72,73]. DAAs may exert potential therapeutic effects on AD through mechanisms involving alleviation of oxidative stress, inhibition of abnormal protein aggregation, and suppression of cholinesterase (ChEs) activity. Xia and colleagues [74] isolated ten DAAs from the mulberry root bark including inethermulberrofuran C, mulberrofuran C, mulberrofuran J, albafuran C, kuwanon G, kuwanon H, mulberrofuran K, mulberrofuran G, isomulberrofuran G, and kuwanol A, as shown in Figure 4. These compounds were systematically evaluated using established in vitro models of AD pathogenesis, with detailed mechanistic analyses presented in subsequent paragraphs [74].

Excessive ROS in the brain can lead to oxidative stress and neuronal death. With oxygen radical absorbance capacity (ORAC) as the determination index and 6-hydroxy-2,5,7,8-tetramethylchroman-2-carboxylic acid (trolox) as the positive control, ten DAAs exhibited antioxidant activities higher than trolox at a uniform testing concentration of 1 μmol·L^−1^. Among them, mulberrofuran C had the strongest antioxidant activity, showing almost 3 times higher activity than Trolox [74].

As mentioned above, one of the main pathological characteristics of AD is the abnormal aggregation of misfolded Aβ polypeptides. Regulating Aβ aggregation is regarded as a promising method for the treatment of AD. With resveratrol (20 µmol·L^−1^) and methylene blue (20 µmol·L^−1^) as positive controls, in thioflavin T fluorescence assay, it was found that the inhibitory activity of compounds mulberrofuran C, mulberrofuran J, albafuran C on Aβ_42_ self-aggregation at the same concentration (20 µmol·L^−1^) were 70%, 56%, and 56%, respectively. Among them, the inhibitory effect of mulberrofuran C was the closest to that of resveratrol (72%) [74]. Tau protein is microtubule-associated protein that is rich in neurons of CNS. In pathological conditions, tau proteins are hyperphosphorylated, resulting in their self-aggregation into neurofibrillary tangles (NFTs). The formation of NFTs can block axonal transport and cause neuronal death [75,76]. The inhibitory activity of these DAAs on tau protein aggregation were detected by a method similar to that used for the inhibition assay of Aβ_42_ self-aggregation. It was found that all ten DAAs exhibited moderate or strong inhibitory activities against tau protein aggregation (35–96%) at the dose of 20 μmol·L^−1^. Among them, the inhibitory activity of kuwanon G on tau protein aggregation (96%) was even higher than that of methylene blue (80%) [74].

In AD patients, the neurotransmitter acetylcholine (ACh) levels at neuronal synapses are reduced [77]. Inhibiting the activity of ChEs including acetylcholinesterase (AChE) and butyrylcholinesterase (BuChE) is desired to maintain normal ACh levels, thereby ensuring the normal function of cholinergic neurons. Compounds isomulberrofuran G, mulberrofuran G, kuwanol A, and mulberrofuran C were found to exhibit potent inhibitory activity on AChE, with half maximal inhibitory concentration (IC_50_) values of 1.4, 2.7, 3.8, 4.2 μmol·L^−1^, respectively [74]. In addition to the inhibitory activity on AChE, studies have revealed the multi-target potential of DDAs against other AD-related enzymes, as exemplified by mulberrofuran G and albanol B (Figure 4) isolated from mulberry root bark [78]. These enzymes include AChE, BuChE, and β-site amyloid precursor protein cleaving enzyme 1 (BACE1), responsible for the production and accumulation of neurotoxic Aβ in the brain. Mulberrofuran G was identified as a mixed-type inhibitor of AChE, BuChE, and BACE1 (IC_50_ = 2.1, 9.7, and 0.3 μmol·L^−1^, respectively). Albanol B demonstrated non-competitive inhibitor against AChE and BuChE (IC_50_ = 2.4 and 1.3 μmol·L^−1^), while acting as a mixed-type inhibitor of BACE-1 (IC_50_ = 0.5 μmol·L^−1^). The dual inhibitory activities on ChEs and BACE1 observed in vitro suggest these compounds may need to be further investigated for AD therapeutic development.

The blood–brain barrier (BBB), a highly selective semipermeable membrane barrier separating the circulating blood from the extracellular fluid of brain tissue, presents a critical bottleneck in the development of neurotherapeutic drugs due to its strict molecular selectivity that prevents over 98% of small-molecule drugs from reaching the CNS [79,80]. Evaluation of BBB permeability using the parallel artificial membrane permeability assay (PAMPA) revealed promising penetration capacities for kuwanon H (4.9 × 10^−6^ cm/s) and mulberrofuran K (8.7 × 10^−6^ cm/s), comparable to some of the clinical drugs. Further, mulberrofuran K was selected to evaluate its protective effect in a nerve injury model. It was found that mulberrofuran K exerted neuroprotective effects by upregulating GSH and inhibiting ROS in a glutamate-induced HT 22 cell model [74]. Notably, despite poor BBB permeability, other DDAs exhibited superior anti-AD effects compared to mulberrofuran K. Therefore, it is necessary to conduct a structure–activity relationship (SAR) study on them. By retaining the core pharmacophore and modifying the auxiliary domains, it is expected to improve the BBB permeability while maintaining the anti-AD efficacy.

### 3.3. Benzofurans

Benzofurans are a class of heterocyclic compounds characterized by the fusion of a benzene ring and a furan ring, widely distributed in higher plants such as Moraceae, Asteraceae, Rutaceae, and Liliaceae. Benzofurans, secondary metabolites of plants, exhibit diverse bioactivities, including antioxidant, anti-inflammatory, antitumor, antibacterial, and anti-AD properties [81].

**Figure 5:**
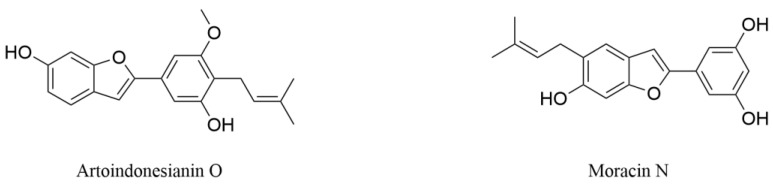
The structures of prominent benzofurans from mulberry with neuroprotective effects.

Artoindonesianin O (AIO, Figure 5), a characteristic benzofuran derivative isolated from mulberry fruit, exhibits multi-target neuroprotective effects against core pathological hallmarks of AD [82]. Through Western blot analysis, it was found that AIO (1–10 μmol·L^−1^) could effectively inhibit the tau hyperphosphorylation induced by okadaic acid (OA). This effect appears mechanistically linked to AIO’s ability to counteract OA-mediated suppression of protein phosphatase 2A (PP2A) activity while modulating extracellular signal-regulated kinase 1/2 (c) signaling cascades. Rat primary hippocampal neurons were treated with cytotoxic oligomeric Aβ_42_ or N-methyl-D-aspartate (NMDA) that induces excitotoxicity to establish in vitro models of AD. AIO treatment significantly reversed the decrease in neuronal viability and ATP synthesis impairment induced by Aβ_42_ or NMDA, with the maximum protective effect at 10 μmol·L^−1^ without detectable cytotoxicity. Notably, this compound demonstrated a unique capacity to promote dendritic spine regeneration in damaged neurons and improve synaptic plasticity [82]. As a multifunctional modulator targeting tau pathology modification, Aβ toxicity mitigation, and synaptic network restoration, AIO represents a novel dietary-derived chemical component that illuminates promising avenues for developing nutritional interventions against AD progression.

Moracin N, isolated from mulberry leaf as shown in Figure 5, exhibited optimal neuroprotective effects by inhibiting ferroptosis in mouse HT22 cells [83]. After treatment with the ferroptosis inducer erastin (0.4 μmol⋅L^−1^), the cell viability of HT22 cells was 17% of the untreated control group. In contrast, after simultaneous addition of moracin N (1–50 μmol⋅L^−1^) with erastin, the viability of HT22 cells accounted for 22–110% of the untreated control group, demonstrating significant anti-ferroptosis activity with half maximal effective concentration (EC_50_) values of 0.4 μmol·L^−1^. Further assessment of the neuroprotective activity mechanism of moracin N revealed that it exerted neuroprotective effects by reducing GSH consumption, inhibiting downregulation of GPx4 in both gene and protein levels, suppressing excessive production and accumulation of ROS and Fe^2+^, and enhancing the activity of antioxidant enzymes [83]. Moracin N exerts neuroprotection through inhibiting ferroptosis, suggesting its potential as a therapeutic strategy for NDs involving ferroptosis, especially acute central nervous system injuries.

### 3.4. Quinones

**Figure 6:**
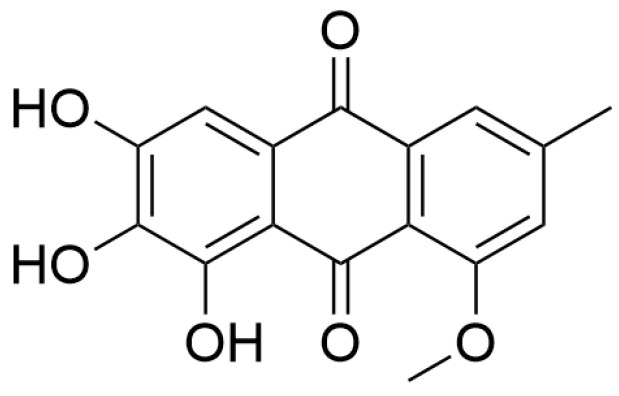
The structure of evariquinone isolated from the endophytic fungus in mulberry.

Evariquinone (Figure 6), a quinone compound isolated from the endophytic fungus (*Colletotrichum* sp. JS-0367) in mulberry, exhibited significant neuroprotective activity in a glutamate-induced HT22 neuronal death model [84]. As established in previous studies, glutamate neurotoxicity arises from dual pathological mechanisms: oxidative stress triggered by ROS overproduction and disruption of ionic homeostasis via pathological calcium influx (Ca^2+^ overload), both of which synergistically activate the mitogen-activated protein kinases (MAPK) signaling pathway [85,86]. It was reported that evariquinone concentration-dependently attenuated glutamate-mediated ROS generation in HT22 cells, achieving maximal inhibitory efficacy at 100 μmol·L^−1^. Meanwhile, it effectively regulated the intracellular Ca^2+^ homeostasis. Consequently, it suppressed phosphorylation cascades of key MAPK pathway components, including c-Jun N-terminal kinase (JNK), extracellular signal-regulated kinase (ERK), and p38 MAP kinase (p38), thereby blocking downstream apoptotic signaling. A structure-activity relationship study revealed that the phenolic hydroxyl groups within its benzoquinone scaffold neutralize free radicals through an electron transfer mechanism, and this property is closely associated with its neuroprotective function [84]. This research breaks through the traditional paradigm of studying active components in mulberry, providing an innovative idea for the design and development of mulberry-based drugs. The exploration of neuroprotective active compounds is not confined to the mulberry itself, other directions and fields related to mulberry also hold potential research value.

### 3.5. Stilbenes

Stilbenes are a class of polyphenolic compounds characterized by the 1,2-diphenylethylene skeleton [87]. They are found in plants such as mulberry, grape, and blueberry as phytoalexins, and can protect plants from biotic and abiotic stresses. These compounds have a variety of biological activities, including antioxidant, anti-inflammatory, antimicrobial, anti-cancer, and anti-obesity [88]. Mulberroside A, resveratrol, and oxyresveratrol are typical stilbene family members, and they have shown therapeutic potential against various NDs.

**Figure 7:**
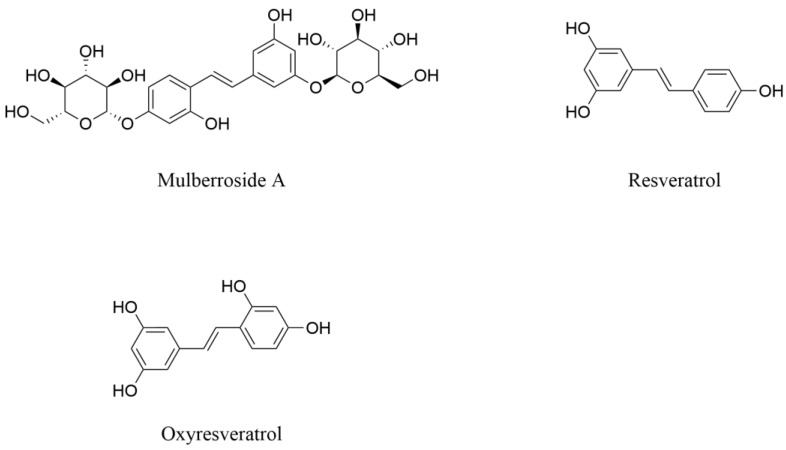
The structures of prominent stilbenes from mulberry with neuroprotective effects.

Mulberroside A (Figure 7) is a kind of polyhydroxy stilbene compound, mainly isolated from the root and branch of mulberry [89]. It exerts beneficial effects through multiple mechanisms, including alleviating oxidative stress, inhibiting neuroinflammation, and repairing internal barriers in nerve injury. Wang and colleagues [90] established a model of ischemic and hypoxic cortical nerve cells in vitro through oxygen–glucose deprivation and reperfusion (OGD/R) treatment. It was found that mulberroside A (20 and 40 μg·mL^−1^) could significantly inhibit the release of LDH, and reduce the proportion of neuronal apoptosis (early and late stages) and necrosis induced by OGD/R in cortical neurons. It was also found that mulberroside A exerted neuroprotective effects by inhibiting the phosphorylation of ERK1/2, JNK1/2, and p38 in the signaling pathway and reducing the levels of pro-neuroinflammatory cytokines. Studies have shown that the brain disorders caused by high-fructose diet (HFrD) are associated with the dysbiosis of intestinal epithelial barrier (IEB) and hippocampal neuroinflammation caused by the damage to the BBB [91]. Mulberroside A significantly ameliorated neuroinflammatory injury induced by HFrD in C57BL/6N male mice. It was found that mulberroside A could remodel IEB homeostasis, increase the content of short-chain fatty acids (SCFAs) in intestinal contents and serum, and reactivate signaling of the colonic NLR family, pyrin domain containing 6 (NLRP6) inflammasome. It also upregulated the expression of mucin 2 (MUC2) and intestinal-type mucus to prevent the damage of IEB, subsequently reducing endotoxin levels in the serum. Additionally, it alleviated oxidative stress damage in the animal models by decreasing SOD and MDA levels, while upregulating the expression of GPX and Nrf2. It also restored BBB integrity through upregulation of tight junction proteins including zonula occludens-1 (ZO-1), occludin, and claudin-5 [92].

Resveratrol (RES, Figure 7), a natural polyphenolic stilbene compound, is widely distributed in plants including grape, tomato, blueberry, jackfruit, and mulberry, with its tissue concentration significantly influenced by species variations and growth conditions [93]. This compound demonstrates therapeutic potential for mitochondrial dysfunction-related NDDs such as AD, PD, Huntington’s disease, and amyotrophic lateral sclerosis through enhancing mitochondrial biogenesis and functional restoration [94]. Beyond NDDs, RES exhibits neuroprotective effects against epileptic neuronal injury. In a kainic acid (KA)-induced rat model of status epilepticus, pre-injection of RES (100 μmol) into the hippocampal CA3 region exerted multidimensional neuroprotective effects. RES effectively counteracted KA-induced ultrastructural damage to mitochondria and dysfunction of respiratory chain complex I. It upregulated key regulators of mitochondrial biogenesis including peroxisome proliferator-activated receptor gamma coactivator 1-alpha (PGC-1α), nuclear respiratory factor 1 (NRF1), mitochondrial transcription factor A (Tfam), enhanced the expression of cytochrome c oxidase 1 (COX1), and increased the content of mitochondrial DNA (mtDNA). Furthermore, RES inhibited epileptic seizure-induced mitochondrial cytochrome c release and reduced caspase-3 activation, consequently attenuating hippocampal neuronal apoptosis [95].

Oxyresveratrol (OXY, Figure 7), structurally similar to RES, is an effective antioxidant [94,96]. It exerts neuroprotective effects through multiple pathways, including anti-oxidative stress, inhibition of inflammation, anti-apoptosis, and regulation of glia functions. As early as 2008, Chao and colleagues [97] treated SH-SY5Y cells with OXY (1–25 μmol·L^−1^) for 30 min, then exposed them to 6-OHDA (25 μmol·L^−1^) for 24 h as injury treatment. It was found that OXY significantly reduced the release of LDH and the activity of caspase-3 in SH-SY5Y cells in a dose-dependent manner. Additionally, the ROS content in SH-SY5Y cells was significantly reduced over time. These findings provide a preliminary understanding of the neuroprotective mechanisms of OXY against 6-OHDA-induced neurotoxicity. In 2012, Weber and colleagues [98] analyzed the neuroprotective effects of OXY by using two in vitro models. They either simulated trauma induced by stretch in co-cultures of neurons and glia or exposed the mouse cortical nerve cells to high concentrations of glutamate (100 μmol·L^−1^). After mild stretch injury of mouse neurons and glia, microtubule-associated protein 2 (MAP-2) was labeled with MAP-2 antibody to reflect the normal morphology of neurons and the number of MAP-2 positive neurons was counted. It was found that the number of MAP-2 positive neurons in the OXY treatment group (50 μmol·L^−1^ or 100 μmol·L^−1^) had no significant difference from untreated control cultures, indicating that OXY had an obvious protective and improving effect on stretch injury. Moreover, in the non-injury treatment group, OXY had no significant effect on the number of MAP-2 positive neurons, indicating there was no neurotoxicity. However, at the same dose, OXY had no protective and improving effect on the damage caused by glutamate. The results suggest that OXY could be used as an improving and protective agent for mild traumatic brain nerve injury. OXY has beneficial effects in specific nerve injuries, but its ineffectiveness in some injuries indicates that its mechanisms of action are selective. Thus, further research on its relationship with different NDs is necessary.

### 3.6. Alkaloids

**Figure 8:**
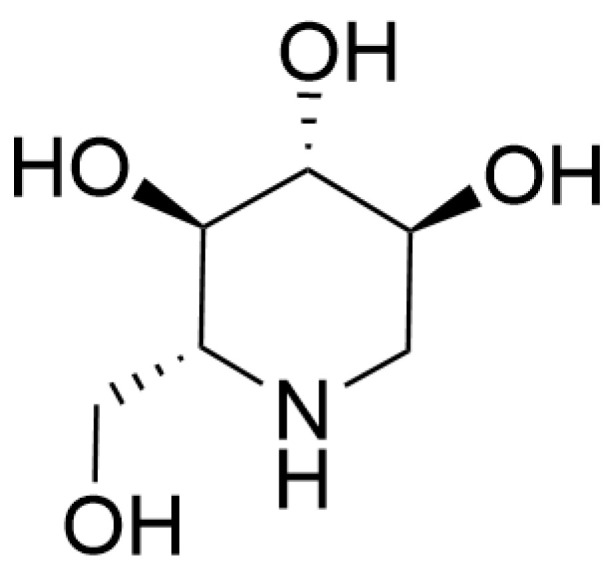
The structure of 1-deoxynojirimycin, a typical alkaloid from mulberry with neuroprotective effects.

DNJ (Figure 8) is naturally present in mulberry tissues, with the highest levels in branches, followed by immature leaves, and minimal levels in mature leaves, requiring chromatographic isolation for purification [99]. DNJ shows beneficial effects on ameliorating the pathological processes related to AD. Chen and colleagues [100] conducted an experimental study using SAMP8 mice (a rapid-aging AD model), administering purified DNJ via oral gavage at 160 mg·kg^−1^·d^−1^ dissolved in distilled water. They discovered that DNJ had several positive effects. Firstly, it significantly enhanced the learning and spatial memory abilities of cognitively impaired SAMP8 mice. It also significantly inhibited Aβ aggregation, microglial activation, and decreased the levels of neuroinflammatory factors (TNF-α, IL-1β and IL-6) in the hippocampus of SAMP8 mice. Furthermore, DNJ revealed that it inhibited Aβ aggregation in SAMP8 mice by suppressing the expression of BACE-1. In another study, DNJ was found to mitigate insulin resistance-induced elevation of phosphorylated tau and Aβ levels [101]. Insulin resistance, a core pathological feature of type 2 diabetes, is closely associated with abnormal tau phosphorylation and Aβ deposition during AD progression [102]. Parida and colleagues [101] established an insulin-resistant model by exposing human SK-N-SH neuroblastoma cells to prolonged hyperinsulinemia. Experimental results demonstrated that insulin-resistant cells treated with DNJ (5 μmol·L^−1^) significantly enhanced insulin receptor (IR)-mediated PI3K/AKT signaling pathway activity. This subsequently suppressed the hyperactivation of glycogen synthase kinase 3β (GSK3β), ultimately reducing pathological tau phosphorylation and inhibiting the formation of NFTs. Notably, DNJ concurrently upregulated the gene of insulin-degrading enzyme (IDE), a key regulatory factor in Aβ clearance. These findings suggest that DNJ is a promising multi-target neuroprotective agent worthy of further research for the development of therapeutics of AD.

In summary, the neuroprotective compounds isolated from mulberry mainly include flavonoids, stilbenes, alkaloids, and other structural classes. These compounds demonstrate protective effects in diverse NDs models, including AD, PD, and cerebral ischemia, through multiple mechanisms of action such as anti-oxidative stress, the suppression of neuroinflammation, and the regulation of apoptotic pathways. We systematically summarized the structural classes, mechanisms of action, applicable disease models, and sources of mulberry parts of these compounds (Table 2), providing critical reference for the discovery of lead compounds, improvement of pharmacological activity, and design of multi-target therapeutics for NDs.

## 4. Limitations and Future Perspective

Despite the demonstrated neuroprotective potential of phytochemicals from mulberry, their practical application remains constrained by some biopharmaceutical limitations. Firstly, the application of mulberry phytochemicals is often hindered by their physicochemical properties, including poor solubility, low bioavailability, short half-life, and instability in biological environments [103]. For example, the high polarity of OXY affects its transmembrane permeability. Once it enters the bloodstream, intestinal metabolism and extensive metabolic conjugation in the liver will rapidly eliminate it, resulting in a short half-life in vivo (about 0.96 h) and low bioavailability (F ∼ 14%) [104,105]. Secondly, most of the phytochemicals with neuroprotective effects are secondary metabolites. They not only exhibit low natural abundance but also exhibit marked seasonal variability [106]. This makes it impossible to extract large amounts of phytochemicals from mulberry for routine bioactivity research and for use in the pharmaceutical or nutraceutical industries [107]. More critically, there is a significant imbalance in current research: as shown in Table 2, the majority of studies are still limited to in vitro models, while the limited in vivo investigations primarily focus on pharmacodynamic evaluation, with pharmacokinetic parameters, particularly the BBB permeability, being neglected. Given that the BBB can exclude over 98% of small-molecule drugs [80], this critical gap in research directly restricts the development of neuroprotective agents. Although the toxicological safety of mulberry extracts has been verified [26,108], the undefined pharmacokinetic profile remains a barrier to clinical translation.

Recent technological innovations have provided multidimensional solutions to overcome the application bottlenecks of phytochemicals from mulberry. In terms of improving bioavailability, nanodelivery systems demonstrate significant advantages: (a) hydrophobic cores or polymeric matrices improve dispersion and solubility of poorly soluble compounds; (b) physical barriers against gastrointestinal protect active molecules from being acids/enzymes degradation; (c) ligand-mediated targeted delivery and transcytosis enhance drug accumulation at pathological sites [109,110]. Research has confirmed that solid lipid nanoparticles (SLN) and nanostructured lipid carriers (NLC) increased the relative bioavailability of OXY to 125% and 177%, respectively [111]. In male Sprague Dawley rats administered with nanoformulations, the average half-life of mulberroside A was prolonged by 236%, and the area under the curve from time zero to time t (AUC_0-t_) increased by 122% [112], highlighting the efficacy-enhancing advantages of nanotechnology. Notably, kaempferol-loaded SLN exhibited enhanced BBB penetration in cerebral ischemia models [113]. Although kaempferol is not mulberry-derived, its delivery mechanism provides valuable insights for CNS targeting of mulberry phytochemicals. Regarding production challenges, while chemical synthesis initially appears viable, the structural complexity of most phytochemicals renders total synthesis commercially unfeasible. Additionally, chemical synthesis processes involving organic solvents, heavy metals, and strong acids/bases raise environmental and safety concerns [107]. In contrast, genetically engineered microbial biosynthesis emerges as a promising alternative. Metabolically engineered *Saccharomyces cerevisiae* ST4140 achieves resveratrol yields of 531 mg/L using ethanol as the carbon source—equivalent to the natural content in 10.6 kg of dried mulberry fruit [114,115]. More importantly, the production of phytochemicals through microbial fermentation can be easily scaled up from the laboratory scale to industrial scale. At the same time, the genetic backgrounds of the microorganisms used (such as *Escherichia coli*, yeast, etc.) have been relatively well studied, facilitating genetic modification [116]. However, it must be pointed out that the existing technologies have not fully resolved the issues of formulation stability and production costs of mulberry phytochemicals. Interdisciplinary research is still needed to advance the clinical translation process.

Plants have traditionally been an important source for the discovery and development of new medicines. The mulberry is not only an economic crop, but also an important medicinal plant, rich in diverse natural active ingredients. With the intensification of population aging, neurological disorders are seriously endangering human health. Their pathogeneses are complex and involve multiple targets or signaling pathways, requiring additional studies for clear understanding of the mechanisms of action. The existing studies have shown that the phytochemicals from mulberry can exert neuroprotective effects through various mechanisms, which have good clinical application prospects and are expected to provide new strategies and new ideas for the treatment of neurological disorders. Additional scientific investigations on the pharmacological efficacy and mechanisms of action of active mulberry phytochemicals will provide opportunities to identify natural products playing a key role in neuroprotection enabling medicinal chemistry efforts to further develop analogs with improved neuroprotective activity. It is noteworthy that advancing in vivo pharmacological evaluations should be coupled with enhanced exploration of pharmacokinetic properties. In particular, a standardized evaluation system for BBB permeability should be established to assess the CNS-targeted delivery efficiency of active phytochemicals.

## Figures and Tables

**Table 1:** Key differences in extraction characteristics between mulberry leaf and fruit.

Comparison Items	Mulberry Leaf	Mulberry Fruit	Refs.
Bioactivity focus	Anti-diabetic, hypolipidemic	Antioxidant, anti-inflammatory	[26,28,29]
Characteristic components	Alkaloids (1-deoxynojirimycin, DNJ), flavonoids	Anthocyanins (cyanidin-3-glucoside, C3G), phenolic acid derivatives	[26,30]
Extraction key points	Enzymatic hydrolysis/ultrasound-assisted cell wall disruption	Light avoidance and temperature control	[31,32,33]
Preferred cultivars	*Morus australis* (higher DNJ content)	*Morus nigra* (higher anthocyanins content)	[34,35]
Optimal harvest period	June–July (DNJ peak)	Full maturity (anthocyanins peak)	[36,37]

**Table 2:** Summary of compounds with neuroprotective effects from mulberry.

Structural Classes	Compound Name	Mechanisms of Action	Disease Models	Plant Parts	Refs.
Flavonoids	Morin	Alleviate oxidative stress	Neuronal injury in vivo model	Branch	[54,56]
		Inhibit inflammation-mediated neuronal apoptosis	PD in vivo model		
	Moralbaflavone C	Alleviate oxidative stress	PD in vitro model	Root bark	[58]
Flavonoids	Sanggenol L	Alleviate oxidative stress and cell apoptosis	PD in vitro model	Root bark	[62]
	Cyanidin-3-glucoside	Maintain mitochondria normal function	Cerebral ischemia in vitro and vivo models	Fruit	[65,66]
	Morachalcone D	Alleviate oxidative stress and inhibit ferroptosis	Neuronal injury in vitro models	Leaf	[70]
Typical Diels-Alder-type adducts	Mulberrofuran C	Alleviate oxidative stress, inhibit aggregation of abnormal protein and activity of cholinesterases	AD in vitro models	Root bark	[74,78]
	Mulberrofuran K				
Benzofurans	Artoindonesianin O	Inhibit aggregation of abnormal protein and promote dendritic spine regeneration	AD in vitro models	Fruit	[82]
	Moracin N	Inhibit ferroptosis	Neuronal injury in vitro model	Leaf	[83]
Quinones	Evariquinone	Alleviate oxidative stress and regulate intracellular Ca^2+^ homeostasis	Neuronal injury in vitro model	Endophytic fungi in leaf	[84]
Stilbenes	Mulberroside A	Alleviate oxidative stress, inhibit neuroinflammation, and repair internal barriers	Metabolic syndrome-associated neuronal injury in vivo model	Root and branch	[92]
	Resveratrol	Promote mitochondrial biogenesis and functional repair, and inhibit neuronal apoptosis	Epileptic neuronal injury in vivo model	Fruit	[94,95]
	Oxyresveratrol	Alleviate oxidative stress, inhibit inflammation and apoptosis, and regulate glia functions	Neuronal injury in vitro models	Branch and fruit	[97,98]
Alkaloids	1-Deoxynojirimycin	Inhibit aggregation of abnormal protein and inflammation	AD in vitro and vivo models	Branch and leaf	[99,100,101]

## Data Availability

No new data were created or analyzed in this study.
